# Cochlear Marginal Cell Pyroptosis Is Induced by Cisplatin *via* NLRP3 Inflammasome Activation

**DOI:** 10.3389/fimmu.2022.823439

**Published:** 2022-04-20

**Authors:** Wenting Yu, Shimin Zong, Peng Zhou, Jiahui Wei, Enhao Wang, Ruijie Ming, Hongjun Xiao

**Affiliations:** Department of Otorhinolaryngology, Union Hospital, Tongji Medical College, Huazhong University of Science and Technology, Wuhan, China

**Keywords:** pyroptosis, cisplatin, marginal cells, NLRP3, TXNIP

## Abstract

Better understanding the mechanism of cisplatin-induced ototoxicity is of great significance for clinical prevention and treatment of cisplatin-related hearing loss. However, the mechanism of cisplatin-induced inflammatory response in cochlear stria vascularis and the mechanism of marginal cell (MC) damage have not been fully clarified. In this study, a stable model of cisplatin-induced MC damage was established *in vitro*, and the results of PCR and Western blotting showed increased expressions of NLRP3, Caspase-1, IL-1β, and GSDMD in MCs. Incomplete cell membranes including many small pores appearing on the membrane were also observed under transmission electron microscopy and scanning electron microscopy. In addition, downregulation of NLRP3 by small interfering RNA can alleviate cisplatin-induced MC pyroptosis, and reducing the expression level of TXNIP possesses the inhibition effect on NLRP3 inflammasome activation and its mediated pyroptosis. Taken together, our results suggest that NLRP3 inflammasome activation may mediate cisplatin-induced MC pyroptosis in cochlear stria vascularis, and TXNIP is a possible upstream regulator, which may be a promising therapeutic target for alleviating cisplatin-induced hearing loss.

## Introduction

Hearing loss is the most common sensory disorder affecting approximately 6.1% of the world population, which can be caused by ototoxic drugs (1–5), excessive noise exposure (6), aging6 (6–10), genetic factors (11–15), and infections (16–18). The ototoxic drug cisplatin is the pioneer of anticancer drugs and mainstay of cancer treatment and has been widely used in the treatment of solid tumors, including ovarian, testicular, and lung cancer (19). However, an actuality that cannot be ignored is that cisplatin can cause severe ototoxic side effects which usually manifested as bilateral progressive irreversible sensorineural hearing loss (20–23). Clinical use of cisplatin often causes hearing loss in 40%–80% of patients (24) and more damage to children (25). However, the exact mechanism of ototoxicity induced by cisplatin remains unclear (26), and there are still no federally approved drugs to prevent or treat cisplatin-induced hearing loss (27, 28).

The stria vascularis (SV), organ of corti (OC), and spiral ganglia are the main areas in cochlea affected by cisplatin (29), of which the most attention has always been paid to the OC in the past (30, 31). Recently, Thomas et al. speculated that marginal cells (MCs) of the SV could be the earliest targets of cisplatin in cochlea (32). Prayuenyong et al. proposed the hypothesis of preferential cochleotoxicity of cisplatin and emphasized the role of SV in the pathogenesis of cisplatin-induced hearing loss (33). Breglio et al. observed the highest cisplatin accumulation in the SV relative to other cochlear regions, whether in mice or humans, and pointed out that SV may be an important intervening target for cisplatin-induced ototoxicity (34). All these results suggested that SV may play a very significant role in the development of cisplatin-induced ototoxicity and should arouse more attention (35). However, few studies reported the pathogenesis of SV in cisplatin-induced hearing loss. Thus, more exploration on SV is necessary, and a better understanding on SV may identify new opportunities for the clinical prevention and treatment of cisplatin-induced hearing loss.

Oxidative stress has been identified as the crucial mechanism of cisplatin-induced ototoxicity in the past 20 years (36–38). However, until now, no antioxidant has been observed to have the effect of mitigating cisplatin-induced hearing loss in clinical trials. Recently, researchers pay increasing attention to the role of inflammation in the pathogenesis of cisplatin-induced ototoxicity (39, 40). Zhang et al. verified that inflammation is involved in cisplatin-induced SV damage (41), but the precise mechanisms involved remained unclear and need further investigation. The NOD-like receptor protein 3 (NLRP3) inflammasome is the most extensively studied inflammasome currently (42). It plays an important role in innate immunity and can promote the repair of injured tissues (43). Previous studies have shown that cisplatin can trigger the assembly of the NLRP3 inflammasome and activate downstream pathways (44, 45); thus, we speculated that the NLRP3 inflammasome may also have a vital effect on the pathogenesis of cisplatin-induced hearing loss.

SV is mainly composed of marginal, intermediate, and basal cells (46), and due to the expression of many ion channels and transporters, MCs are thought to be the most important component for the overall function of SV (47). In this study, we established an *in vitro* model of cisplatin-induced MC damage and demonstrated that cochlear marginal cell pyroptosis is induced by cisplatin *via* NLRP3 inflammasome activation, which may provide a novel intervention target for the prevention and treatment of cisplatin-induced hearing loss.

## Materials and Methods

### Primary MC Culture and Identification

All experimental procedures in this study were performed according to the National Institutes of Health Guidelines for the Care and Use of Laboratory Animals (NIH Publication No. 8023, revised 1978) with the approval of the Animal Care Committee of Tongji Medical College of Huazhong University of Science and Technology.

SV of the cochlea was isolated from Sprague-Dawley (SD) rats at postnatal day 3 and digested with 0.1% type II collagenase (Sigma-Aldrich, St. Louis, MO, USA) for 30 min at 37°C (48, 49). After centrifugation for 5 min at 1,000 rpm, the cells were resuspended in Epithelial Cell Medium-animal (EpiCM-animal, ScienCell, Carlsbad, CA, USA) and cultured at 37°C with 5% CO_2_. Primary MCs were identified by immunofluorescence staining of cytokeratin-18, which was a characteristic molecule of MCs.

### Cell Viability Assay

The MCs cultured in 96-well plates were treated with different concentrations of cisplatin (0~1,000 μM) for 24, 48, and 72 h, respectively. Cell viability was detected using the Cell Counting Kit-8 (CK04, Dojindo, Rockville, MD, USA) at a different time point after incubation according to the manufacturer’s instructions.

### Small Interfering RNA Transfection

**Figure 1:**
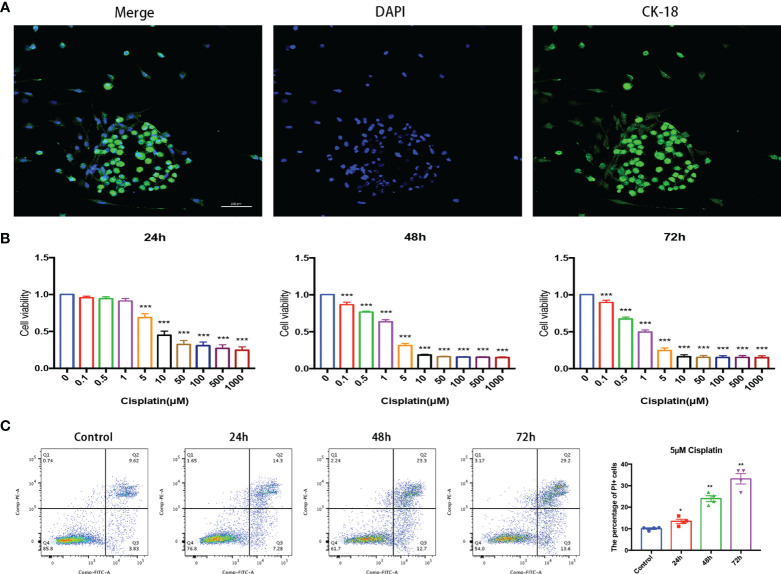
Cisplatin induced MC damage *in vitro* in a time- and concentration-dependent pattern. **(A)** MCs grow in clumps and express CK-18 characteristically. Scale bar: 200 µm. **(B)** Cell viability of MCs under cisplatin treatment at gradient concentrations of 0, 0.1, 0.5, 1, 5, 10, 50, 100, 500, and 1,000 (μmol/L) for 24, 48, and 72 h, respectively. **(C)** Representative images that cisplatin induced the variation of the proportion of MCs with membrane rupture (PI+, the percentage of cells in Q1 and Q2 quadrant) with the time prolongation detected by flow cytometry. The control group was not treated with cisplatin, and the other groups were treated with 5 μmol/l cisplatin for 24, 48, and 72 h, respectively. The above experiment was repeated at least 3 times. *p < 0.05, **p < 0.01, ***p < 0.001.

To knock down the expression of NLRP3 or thioredoxin-interacting protein (TXNIP), MCs were transfected with NLRP3-small interfering RNA (siRNA) or TXNIP-siRNA (RiboBio, Co., Ltd., Guangzhou, China) with Lipo3000 (Invitrogen, Carlsbad, CA, USA, L3000-015) according to the manufacturer’s instructions. MCs transfected with NC-siRNA (RiboBio. Co., Ltd., China) were taken to be the transfection control (siNC). The untreated MCs were set as normal control. The subsequent experiments were performed 24 h after transfection completion. The sequences of each siRNA are listed in **Supplementary Table 1**. We designed three small interfering RNA sequences targeting NLRP3 and TXNIP and selected the siRNA sequence with the highest interference efficiency for subsequent transfection (**Supplementary Figure 1** and **Figure 1**).

### Cell Death Detection by Flow Cytometry

The cell death ratios were determined by flow cytometry with a FITC/Annexin V kit (556547, BD Biosciences) according to the manufacturer’s instructions. Briefly, MCs were harvested *via* trypsinization after treatment as predesigned, then rinsed twice with cold PBS and resuspended in 200 μl of 1× binding buffer. Isolated single-cell suspensions were stained with 5 μl of Annexin V-FITC and 5 μl of PI for 10 min at 37°C in the dark. Finally, the stained MCs were determined *via* flow cytometry (FACSCalibur, BD Biosciences, Carlsbad, CA, USA).

### Real-Time PCR

The total RNA of MCs was extracted with TRIzol reagent (Takara, Kusatsu, Japan) and reverse transcribed to cDNA using the PrimeScript RT Master Mix Kit (Takara, Kusatsu, Japan) according to the manufacturer’s instructions. RT-PCR reaction was performed using a real-time PCR system (Applied Biosystems, Foster City, CA, USA). The mRNA expression was normalized to GAPDH, and the results were calculated using the comparative cycle threshold (ΔΔCt) method. The sequences of primers are listed in the **Supplementary Table 2**.

### Western Blotting Analysis

The total protein of MCs was extracted with RIPA lysis buffer. The protein samples were subjected to SDS-PAGE (8%–10%) separation and bound to the polyvinylidene difluoride membrane. Then the protein bands were blocked with 5% skim milk and incubated with a primary antibody against NLRP3 (A5652, 1;1,000, ABclonal Technology, Woburn, MA, USA), ASC (bs-6741R, 1:1,000, Bioss, Woburn, MA, USA), caspase-1 (GB11383, 1:1,000, Servicebio, Wuhan, China), TXNIP (14715, 1:1,000, Cell Signaling Technology, Danvers, MA, USA), GSDMD (AF4012, 1:1,000, Affinity Biosciences, Cincinnati, OH, USA), GAPDH (20770-1-AP, 1:1000, Proteintech, Wuhan, China), N-GSDMD (ab215203,1:1000, Abcam, Cambridge, MA, USA), and cleaved-caspase1 (sc-398715, 1:200, Santa Cruz, Dallas, TX, USA) overnight at 4°C. The horseradish peroxidase-labeled secondary antibody and ECL detection kit were used to detect the target proteins. The ratio of the gray value of the target protein to the corresponding GAPDH was the relative expression of the target protein.

### Immunofluorescence

MCs were cultured in confocal dishes and treated as predesigned. The cells were washed with PBS and fixed with 4% paraformaldehyde for 30 min at 37°C. Then, the cells were washed with PBS and permeabilized with 0.3% Triton X-100 for 20 min at room temperature.

For TUNEL staining, the cells were washed after permeabilization, then commercial TUNEL Kits (40308ES60, Yeasen, Shanghai, China) were used to detect cells with fractured DNA according to the manufacturer’s instructions.

For the detection of target proteins, the cells were washed after permeabilization, blocked with donkey serum (AntGene, Wuhan, China) for 30 min at room temperature, then incubated with a primary antibody against CK-18 (abs123946, 1:400, Absin Bioscience, Shanghai, China), NLRP3 (NBP2-12446, 1:100, Novus Biologicals, Littleton, CO, USA), ASC (ab175449, 1:100, Abcam, Cambridge, MA, USA), caspase-1 (sc-398715, 1:100, Santa Cruz, Dallas, TX, USA), thioredoxin-interacting protein (TXNIP, ab210826, 1:300, Abcam), and GSDMD (20770-1-AP, 1:500, Proteintech) overnight at 4°C. Then the cells were washed with PBST and incubated with fluorochrome-conjugated secondary antibody (1:500, AntGene, Wuhan, China) for 1 h at 37°C. Subsequently, the cells were washed with PBST and counterstained with DAPI (AntGene, Wuhan, China) for nuclei for 5 min. Finally, the cells were observed with a laser-scanning confocal microscope (Nikon, Tokyo, Japan).

### Enzyme-Linked Immunosorbent Assay

IL-1β levels in the culture supernatant of primary MCs were determined by the commercial ELISA kit (RLB00, R&D Systems, Minneapolis, MN, USA); the testing process was carried out according to the manufacturer’s instructions. Optical density was read at 450 nm using a microplate reader.

### Cell Ultrastructure Observation Under Electron Microscope

After treatment as predesigned, the MCs were immediately soaked in a commercial electron microscope fixing solution at room temperature for 2 h, and then soaked in 1% osmium acid for 2 h at dark-room temperature. After alcohol shaving and dehydration, the cells were permeated and embedded, and then ultrathin slices were made and stained with alcoholic uranyl acetate and alkaline lead citrate. After cleaning and drying, the slices were observed using the transmission electron microscope (TEM, HT7800/HT7700, Hitachi, Tokyo, Japan).

For scanning electron microscope (SEM) observation, the cells were postfixed with 1% of OsO_4_ at room temperature in the dark for 2 h. Then they were dehydrated through ethanol series and critical-point dried, mounted on stubs, and sputter-coated with a thin layer of conductive metal, gold, and palladium; finally, the images were observed under SEM (TEM, HT7800/HT7700, Hitachi, Tokyo, Japan).

### Statistical analysis

All experiments were performed independently at least in triplicate, and data were presented in the form of mean ± standard error. Statistical analysis was conducted by GraphPad Prism 7.0 software. Two-tailed Student’s t-tests were used to compare the data means between two groups. The means of more than two groups were compared using a one-way ANOVA. A value of p < 0.05 was considered statistically significant.

## Results

### Cisplatin Induced MC Damage *In Vitro* in a Time- and Concentration-Dependent Manner and Increased the Expression of NLRP3 in MCs

Under a fluorescence microscope, the primary cultured MCs grew in clusters and specifically expressed CK-18, which was significantly different from non-MCs (**Figure 1A**).

The results of CCK-8 showed that the cell viability of MCs decreased with the increase in cisplatin concentration regardless of whether the time of cisplatin treatment was 24, 48, or 72 h (**Figure 1B**). The results of flow cytometry implied that under the treatment of 5 μM cisplatin, the proportion of MCs with membrane rupture (PI+, the percentage of cells in the Q1 and Q2 quadrants) increased with the prolongation of cisplatin treatment (**Figure 1C**).

**Figure 2:**
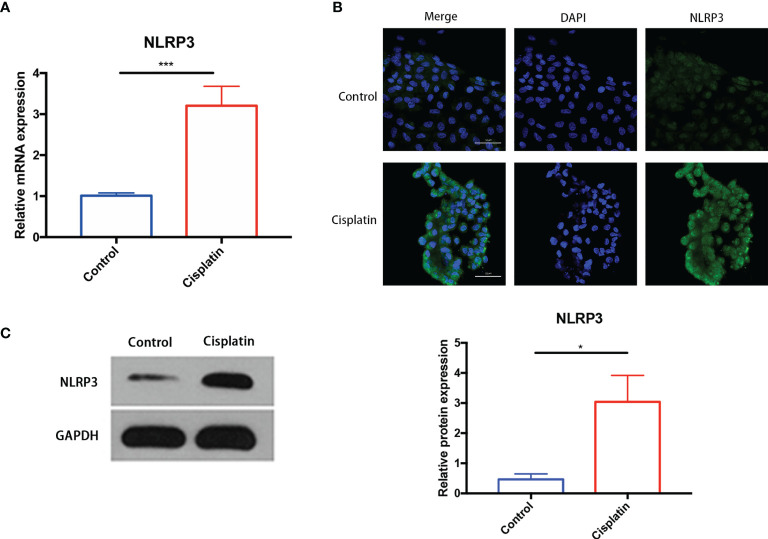
Cisplatin induced an increased expression of NLRP3 in MCs at both the mRNA level and the protein level. **(A)** The expression difference of NLRP3 mRNA in MCs between the control and the cisplatin group (5 μmol/l cisplatin for 24 h). **(B, C)** The increased expression of NLRP3 protein in the cisplatin group compared with the control detected by immunofluorescence **(B)** and Western blotting **(C)**. Green fluorescence indicates FITC-labeled NLRP3 protein, and blue fluorescence indicates DAPI-labeled nuclei. Scale bars: 50 µm. Representative results of at least three repeated experiments are shown. *p < 0.05, ***p < 0.001.

The results of PCR, Western blot, and immunofluorescence indicated that, compared with the control group, the expression of NLRP3 in cisplatin MCs increased significantly at both the mRNA level and the protein level (**Figure 2**).

### Downregulation of NLRP3 Decreased the Number of MCs With Cell Membrane Rupture or DNA Rupture

**Figure 3:**
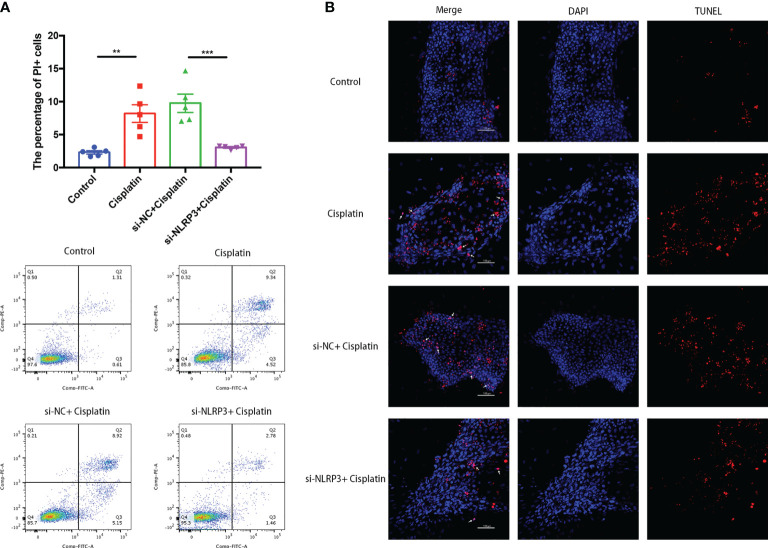
Downregulation of NLRP3 decreased the number of MCs with cell membrane rupture **(A)** or DNA rupture **(B)**. **(A)** The differences in the percentage of MCs with cell membrane rupture (the cells in the Q1 and Q2 quadrants) between the groups. **(B)** The differences in the percentage of MCs with DNA fracture (TUNEL positive) between the groups. Blue fluorescence indicates DAPI-labeled cell nuclei, and red fluorescence indicates DNA fragmentation labeled with Alexa Fluor 640. The red fluorescence overlapping with the nucleus was judged to be TUNEL-positive cells (white arrows). Scale bar: 100 µm. Representative results of at least three repeated experiments are shown. **p < 0.01, ***p < 0.001.

As shown in **Figure 3A**, the percentage of MCs with cell membrane rupture (the cells in the Q1 and Q2 quadrants) in the cisplatin group (8.19 ± 1.33%) was higher than that in the control group (2.3 ± 0.25%). However, NLRP3-siRNA significantly reduced the percentage compared with the cisplatin+siNC group (cisplatin+siNLRP3 group: 3.07 ± 0.12%, cisplatin+siNC group: 9.76 ± 1.39%). The results of TUNEL staining are shown in **Figure 3B**, which show that the amount of TUNEL-positive cells in the cisplatin+siNLRP3 group is relatively small than that in the cisplatin+siNC group. This difference is similar to the difference in the number of cells with ruptured cell membranes.

### NLRP3-siRNA Attenuates the Cisplatin-Induced Pyroptosis-Like Morphological Changes on the Cell Membrane of MCs

**Figure 4:**
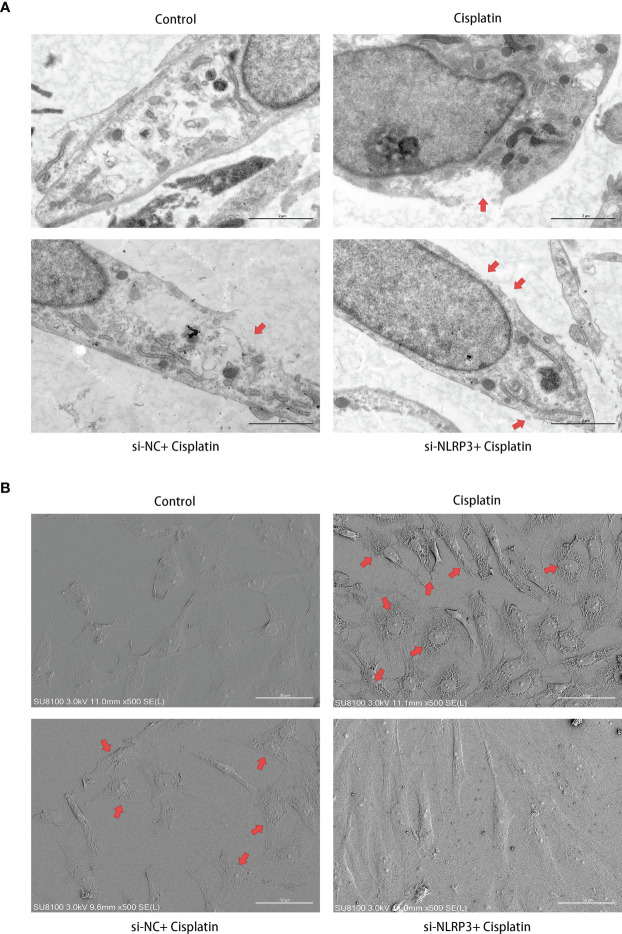
Downregulation of NLRP3 reversed the cisplatin-induced ultramicroscopic changes in MCs. **(A)** The morphologic changes of MCs after different treatments under TEM. The red arrows indicate the ruptured cell membrane and local lighter-stained cytoplasm. Scale bars: 2 µm. **(B)** The morphological changes of MCs observed by SEM. The red arrows indicate the withered tree-like cell membrane at the edge of the cell. Scale bars: 50 µm. Representative results of at least three repeated experiments are shown.

As shown in **Figure 4A**, under the TEM, the cell membrane of MCs in the control group was continuous and smooth, while the cell membrane of MCs in the cisplatin group showed local rupture. The cytoplasm near the rupture showed low-density staining, which seemed to be local swelling-related rupture. The cells in the cisplatin+siNC group showed morphological changes similar to those in the cisplatin group. In the cisplatin+siNLRP3 group, although there were a few of small discontinuities in the MCs’ membrane, there was no obvious low-density staining at the nearby cytoplasm, and no obvious signs of local swelling-related rupture were found.

As shown in **Figure 4B**, different from the control group under the SEM, the MCs’ membrane in the cisplatin group showed larger hole-like changes. The cell membrane resembled withered tree branches. The above changes were more obvious at the cell edges. In the MCs of the siNLRP3 group, the above changes disappeared.

### Downregulation of NLRP3 Inhibits the Expression of Key Molecules in the Process of Cisplatin-Induced NLRP3 Inflammasome-Mediated Inflammatory Programmed MC Death (Pyrolysis)

**Figure 5:**
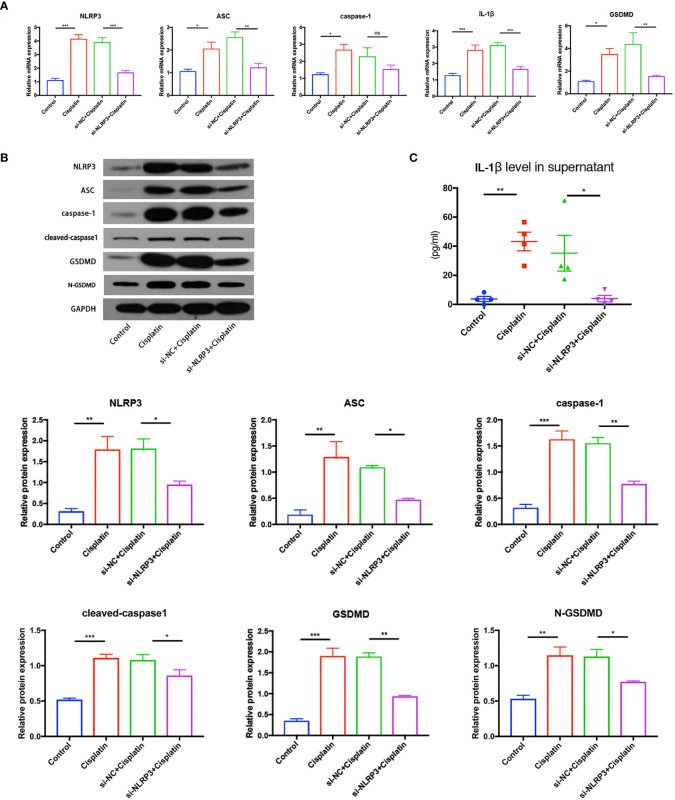
The expression differences of key molecules in NLRP3 inflammasome-mediated pyrolysis (NLRP3, ASC, caspase-1, IL-1β, and GSDMD) in mRNA level **(A)** and in protein level **(B)** in the control, cisplatin, cisplatin+siNC, and cisplatin+siNLRP3 groups. **(C)** The difference in the IL-1β concentrations in the supernatant of each group. Representative results of at least three repeated experiments are shown. *p < 0.05, **p < 0.01, ***p < 0.001. ns, no significance.

**Figure 6:**
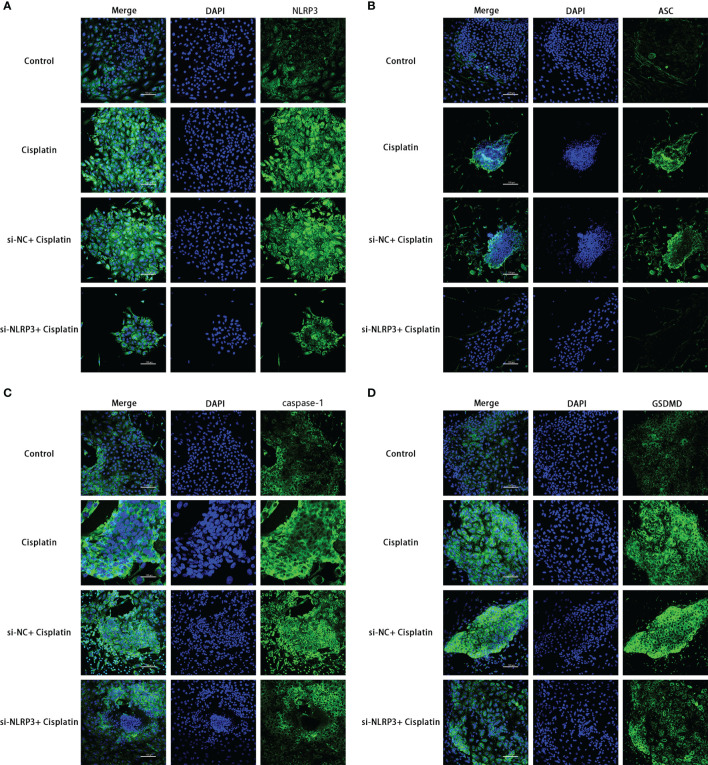
The expression differences of NLRP3, ASC, caspase-1, and GSDMD in the control, cisplatin, cisplatin+siNC, and cisplatin+siNLRP3 groups. Blue fluorescence indicates DAPI-labeled cell nuclei, and green fluorescence indicates FITC-labeled target proteins. Representative results of at least three repeated experiments are shown. Scale bar: 100 µm.

The results of RT-PCR, Western blotting, and immunofluorescence show that cisplatin caused an increased expression of NLRP3, ASC, caspase-1, and GSDMD at both mRNA and protein levels. The expression level of the above molecules in the siNLRP3 group was significantly lower than that in the cisplatin group (**Figures 5A, B**, **6**).

As shown in **Figure 5C**, the concentration of IL-1β in the supernatant of the cisplatin group (43.25 ± 6.391 pg/ml) was higher than that of the control group (3.774 ± 1.727 pg/ml), and the concentration in the cisplatin+siNLRP3 group (4.025 ± 2.233 pg/ml) was significantly lower than that in the cisplatin+siNC group. All differences are statistically significant (p < 0.05). These findings indicate that cisplatin may activate NLRP3 inflammasome-mediated pyroptosis in MCs, whereas downregulation of NLRP3 could inhibit cisplatin-induced MC pyroptosis.

### Downregulating TXNIP Can Inhibit Cisplatin-Induced NLRP3 Inflammasome-Mediated Pyroptosis in MCs

**Figure 7:**
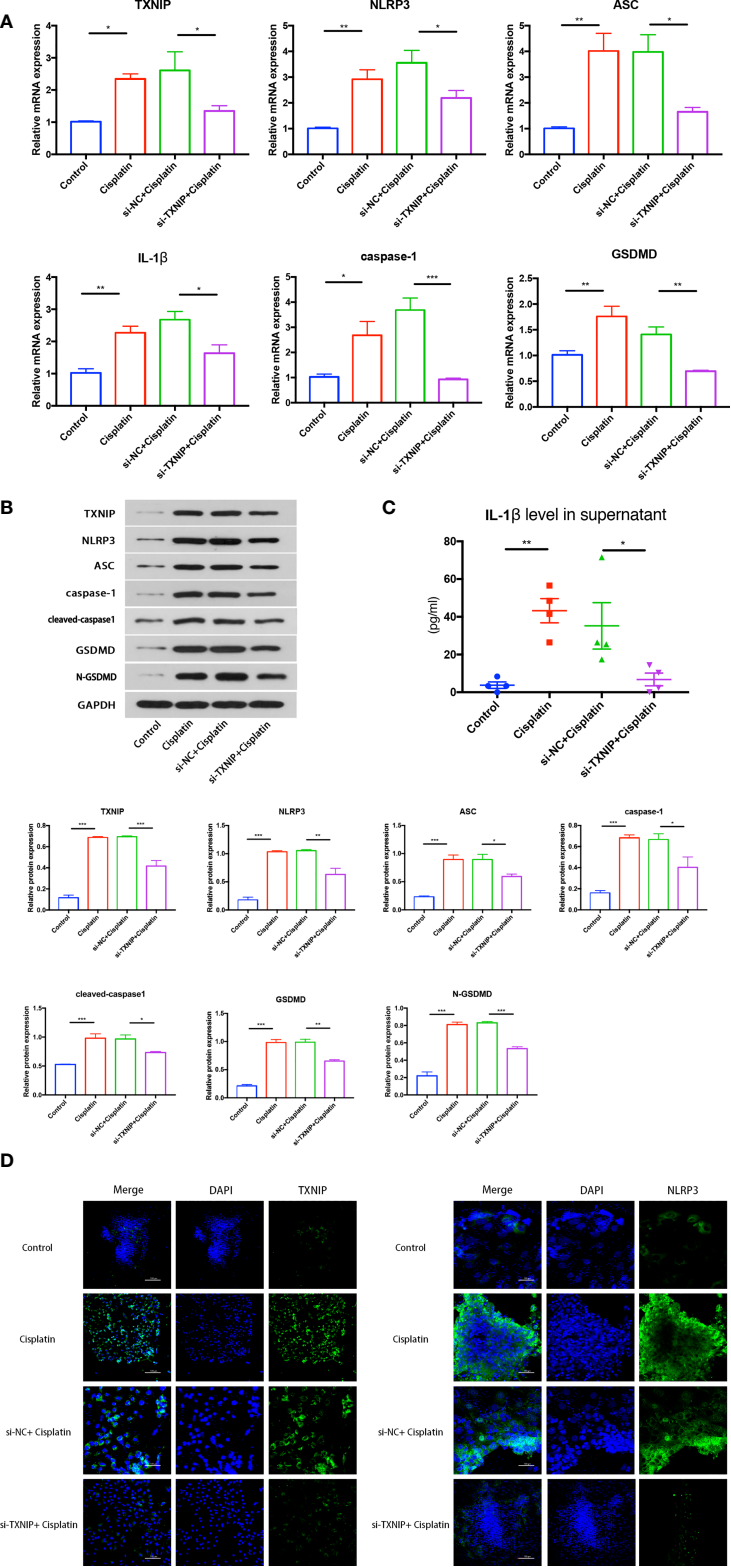
The effect of downregulation of TXNIP on cisplatin-induced NLRP3 inflammasome-mediated pyrolysis-related protein expression in MCs at mRNA level **(A)** and protein level **(B)**. **(C)** The difference in the concentrations of IL-1β in the supernatant in each group. **(D)** The variation of NLRP3 expression before and after TXNIP-siRNA transfection. Scale bars: 100 µm. Representative results of at least three repeated experiments are shown. *p < 0.05, **p < 0.01, ***p < 0.001.

The expression difference of NLRP3 inflammasome-mediated pyroptosis-related proteins in the control, cisplatin, cisplatin+siNC, and cisplatin+siTXNIP groups at mRNA and protein levels is shown in **Figures 7A, B**. The expressions of TXNIP, NLRP3, ASC, caspase-1, IL-1β, and GSDMD in the siTXNIP group were all lower than those in the siNC group at both mRNA and protein levels. As shown in **Figure 7C**, the concentrations of IL-1β in the control, cisplatin, cisplatin+siNC group, and cisplatin+siTXNIP transfection groups were 3.774 ± 1.727, 43.25 ± 6.391, 35.22 ± 12.29, and 6.784 ± 3.373 pg/ml, respectively. As shown in **Figure 7D**, the expression of NLRP3 in the cisplatin+siTXNIP group is significantly lower than that in the cisplatin+siNC group.

### Downregulation TXNIP Can Suppress Cisplatin-Induced MCs’ Membrane Rupture and DNA Fracture Formation

**Figure 8:**
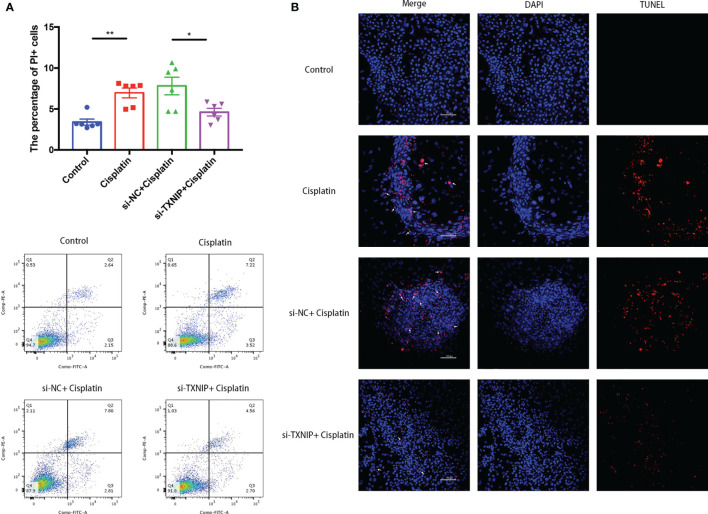
Downregulation of TXNIP decreased the number of MCs with cell membrane rupture **(A)** or DNA rupture **(B)**. **(A)** The differences in the percentage of MCs with cell membrane rupture (the cells in the Q1 and Q2 quadrants) between the groups. **(B)** The differences in the percentage of MCs with DNA fracture (TUNEL positive) between the groups. Blue fluorescence indicates DAPI-labeled cell nuclei, and red fluorescence indicates DNA fragmentation labeled with Alexa Fluor 640. The red fluorescence overlapping with the nucleus was judged to be TUNEL-positive cells (white arrows). Scale bars: 100 µm. Representative results of at least three repeated experiments are shown. *p < 0.05, **p < 0.01.

The percentage of MCs with membrane rupture in the cisplatin+siTXNIP group (4.61 ± 0.47%) is significantly lower than that in the cisplatin+siNC group (7.8 ± 1.08%) (**Figure 8A**). Similarly, the difference in the amount of TUNEL-positive cells in the cisplatin+siTXNIP group is obviously fewer than that in the cisplatin+siNC group (**Figure 8B**).

**Figure 9:**
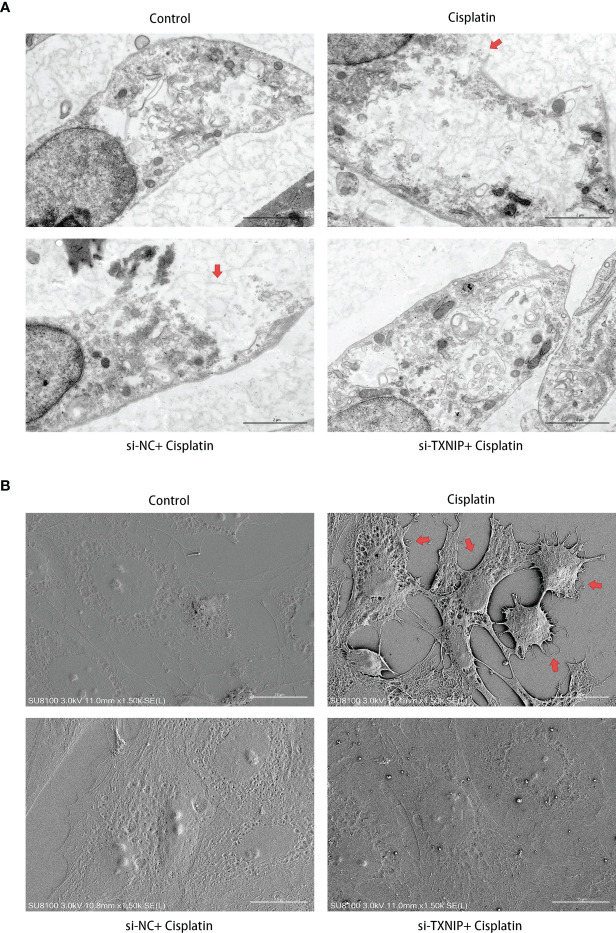
Downregulation of TXNIP reversed the cisplatin-induced ultramicroscopic changes in MCs, similar to that after downregulating NLRP3. **(A)** The morphologic changes of MCs after different treatments were evaluated under TEM. The red arrows indicate the ruptured cell membrane and local lighter-stained cytoplasm. Scale bars: 2 µm. **(B)** The morphological changes of MCs were observed by SEM. The red arrows indicate the withered tree-like cell membrane at the edge of the cell. Scale bars: 15 µm. Representative results of at least three repeated experiments are shown.

The morphology changes of MCs under TEM and SEM are shown in **Figure 9**. The ultrastructure of MCs in the cisplatin+siNC group is similar to that in the cisplatin group which was characterized by the local ruptured cell membrane and withered branch-like cytoplasm. However, TXNIP-siRNA, like the NLRP3-siRNA, alleviates the above pyroptosis-related ultra-morphological changes of MCs caused by cisplatin.

## Discussion

In recent years, inflammatory responses have gradually become research hotspots in the field of sensorineural hearing loss and have received extensive attention from researchers. Previous studies have implied that cisplatin showed a significant pro-inflammatory effect with significantly elevated levels of inflammatory cytokines such as IL-1β, TNF-α, IL-10, and IL-6 (50–53). Therefore, it is reasonable to speculate that inflammatory signals may also be involved in cisplatin-induced SV damage and hearing loss (54, 55). The results of this study demonstrate that NLRP3 inflammasome-mediated inflammatory programmed cell death (pyroptosis) is one of the possible reasons for cisplatin-induced MCs damage, and TXNIP may be the upstream signal that activates the NLRP3 inflammasome.

The NLRP3 inflammasome is composed of innate immune receptor protein NLRP3, adaptor protein ASC, and inflammatory protease caspase-1 (56). It is one of the important initiation signals of inflammation. In this study, the increased expression of NLRP3, ASC, and caspase-1 implied that cisplatin may stimulate the elevated expression of the components of the NLRP3 inflammasome in MCs. It is worth noting that caspase-1 is primarily responsible for the maturation of pro-IL-1β into its biologically active form, and IL-1β is a key cytokine that mediates inflammation and coordinates innate and adaptive immune responses (57, 58). Thus, the increased expression of IL-1β in both intracellular and cultured supernatant in our results suggests that cisplatin may indeed promote the assembly of the NLRP3 inflammasome in MC and induces the expression and secretion of IL-1β through NLRP3 inflammasome activation. In addition, in the rats’ cochlea tissue of cisplatin-induced ototoxicity, we also observed an increase in the expression of NLRP3 in the MC layer, which indicates that the same or similar changes may exist *in vivo* (**Supplementary Figure 3**).

Pyroptosis is a form of inflammatory programmed cell death and is characterized by cell swelling, pore formation in the local plasma membrane, DNA fracture, and GSDM expression (59, 60). GSDMD is activated by inflammatory caspases and cleaved into the n-terminal and C-terminal, where the n-terminal forms a transmembrane pore that releases cytokines, ultimately leading to intense inflammation and cell death (61, 62). Therefore, GSDMD was identified as the executor of pyroptosis (63, 64). After cisplatin treatment, we detected an increased expression of GSDMD in MCs. Meanwhile, we observed that cells were deformed, the cell membrane was incomplete, and many small pores appeared on the membrane under TEM and SEM. This is consistent with the previous results described by other researchers that inflammatory signals activated GSDMD, which caused pyroptosis-forming pores that impair cell membrane integrity (65–67). Although there was a slight difference in the GSDMD pore diameter from the uniform 15–32 nm reported by Zeng et al. (68), it may be due to the different cell types. In addition, downregulating NLRP3 can reduce the percentage of damaged marginal cells under cisplatin treatment as well as the expression levels of GSDMD and IL-1β. Therefore, it is reasonable to speculate that the cisplatin-induced MC pyroptosis may be mediated by the NLRP3 inflammasome and its downstream inflammatory signals.

TXNIP is the main binding medium of the thioredoxin (TXN) antioxidant system (69, 70). Previous studies have shown that TXNIP is involved in cell death mediated by the inflammatory signaling pathway (71, 72). However, the exact mechanisms have not been clarified. In this study, downregulating TXNIP can inhibit the NLRP3 inflammasome-mediated MC pyroptosis under cisplatin treatment, which implied that TXNIP may be the upstream factor of NLRP3 inflammasome activation. It is worth noting that TXNIP could bind to NLRP3 in a redox-dependent manner, thereby promoting the release of IL-1β and subsequent inflammation (73, 74). In addition, TXNIP can inhibit the antioxidant effect of thioredoxin (TRX) by binding to TRX and promote the production and accumulation of ROS (75, 76). Being an upstream factor of both oxidative stress and inflammation, TXNIP is thought to act as a molecular link between these different mechanisms in disease progression. On the other hand, in the MCs under the cisplatin treatment, is there a regulatory factor more upstream on TXNIP? How does cisplatin affect the expression level of TXNIP? These are issues worthy of further study. According to existing reports, psychopathological events such as oxidative stress and endoplasmic reticulum stress may be the cause of the increase in TXNIP expression induced by cisplatin (77–79). Furthermore, a recent analysis of cisplatin resistance has confirmed that TXNIP is a downstream target molecule of UCA1 (80). UCA1 is an lncRNA that has been confirmed to play a role in cisplatin pharmacology by regulating Wnt/β-catenin, Mir-143/FOSL2, Mir-495/NRF2, and other pathways (81–83). TXNIP has also been reported to play different roles by binding to different microRNAs (84–86). However, these studies are piecemeal and have not yet formed a concrete network. In summary, we consider that the cisplatin/LncRNA/microRNA/TXNIP axis is promising upstream pathways.

There are some limitations to our experiment. Firstly, the growth rate of primary MCs was slow and it could not grow limitlessly, and the optimal culture time was observed to be 5–6 days. Considering the time needed for transfection and recovery (usually 12–24 h), the time of cisplatin treatment has to be no longer than 24–36 h, so the time was finally set to 24 h. Therefore, the results of this study can only represent the injury mechanism of MCs under cisplatin treatment in a relatively short time with high concentration, and it cannot be ruled out that different mechanisms may exist in cisplatin-induced MC damage with a small dose for a long time. Secondly, due to the lack of effective vectors for specific transfection to SV in animals, our experiments were conducted on primary MC models *in vitro*. In the future, the realization of targeted interventions on specific targets of MCs in animal experiments will further enhance the level of evidence and persuasiveness of the research. Thirdly, due to the lack of a reliable quantitative detection method for the proportion of pyrolytic cells, we cannot deny the existence of apoptosis or other cell death modes in the model of this study. However, which cell death mode is the most important death mode of MCs under cisplatin treatment? Is there a mutual transition in the process of different death modes, and what are the transition conditions? These issues need to be further studied in the future. Finally, whether inhibition of the NLRP3 inflammasome can relieve the anticancer effect of cisplatin remains to be verified and weighed, local cochlear drug delivery may be a promising solution to this problem in the future.

**Figure 10:**
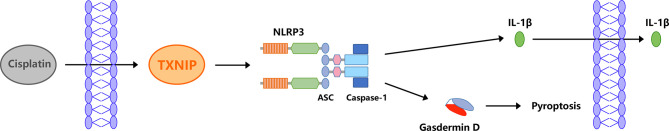
The schematic diagram of cisplatin-induced NLRP3 inflammasome-mediated MC pyroptosis.

In summary, the results of this studies suggest that NLRP3 inflammasome-mediated pyroptosis may be one of the mechanisms involved in cisplatin-induced MC damage, and TXNIP may be the upstream signal that activates the NLRP3 inflammasome (**Figure 10**). It can be inferred that NLRP3 may be a key target of cisplatin-induced damage to the MCs in the cochlea. We expect that these findings would contribute to the prevention and treatment of cisplatin-induced ototoxicity.

## Data Availability Statement

The raw data supporting the conclusions of this article will be made available by the authors, without undue reservation.

## Ethics Statement

The animal study was reviewed and approved by the Institutional Animal Care and Use Committee Tongji Medical College, Huazhong University of Science and Technology.

## Author Contributions

WY and SZ contributed to conception and design of the study. WY performed the experiments and plotted the data. WY and SZ wrote the first draft of the manuscript. SZ revised the manuscript. HX funded and supervised the project. All authors contributed to the manuscript revision and read and approved the submitted version.

## Funding

This work was supported by the National Natural Science Foundation of China (Grant numbers 81771002 and 82071057).

## Conflict of Interest

The authors declare that the research was conducted in the absence of any commercial or financial relationships that could be construed as a potential conflict of interest.

## Publisher’s Note

All claims expressed in this article are solely those of the authors and do not necessarily represent those of their affiliated organizations, or those of the publisher, the editors and the reviewers. Any product that may be evaluated in this article, or claim that may be made by its manufacturer, is not guaranteed or endorsed by the publisher.
