# Supplementary material for: Cochlear Marginal Cell Pyroptosis Is Induced by Cisplatin via NLRP3 Inflammasome Activation
Source: Front Immunol. 2022 Apr 20;13:823439. doi: 10.3389/fimmu.2022.823439 (PMC9067579; doi:10.3389/fimmu.2022.823439)
Supplement: Supplementary file 1 [file DataSheet_1.pdf]

frontiers

Supplementary Material

frontiers

Supplementary Table 1. The siRNA sequences used in transfection.

		Target sequence
si-NLRP3	#1	GCTTCAGCCACATGACTTT
	#2	CCCTGGGATTTCTCCACAA
	#3	GCAATGCCCTTGGAGACAT
si-TXNIP	#1	UGGUCACGUCGAAAUGAAUTT
	#2	AUCCCUUCUAUAUAGGCACCUGUGUC
	#3	GGACUACUUGCGCUAUGAATT

Supplementary Table 2. The primer sequences used in real-time quantitative PCR.

Gene	Primer sequence	
	Forward (5'-3')	Reverse (5'-3')
NLRP3	ATGAGTGTTCGCTGCAAGCTG	TCGCCTGTTGATCGCTGCAAA
ASC	TTGCTGGATGCTCTGTATGG	CCAAGTAGGGCTGTGTTTGC

Caspase−1	GCAGCACAGACTTTCAACATC	GCAGCAGCAACTTCATTTCTC
IL-1β	TGCTGTCTGACCCATGTGAG	GTCGTTGCTTGTCTCTCCTTG
GSDMD	CCAACATCTCAGGGCCCCAT	TGGCAAGTTTCTGCCCTGGA
TXNIP	GTGAAGTTACCCGAGTCAAAGC	CTCACCTGTAGGCTGGTCTTCT
GAPDH	GAAGGTCGGTGTGAACGGAT	CCCATTTGATGTTAGCGGGAT

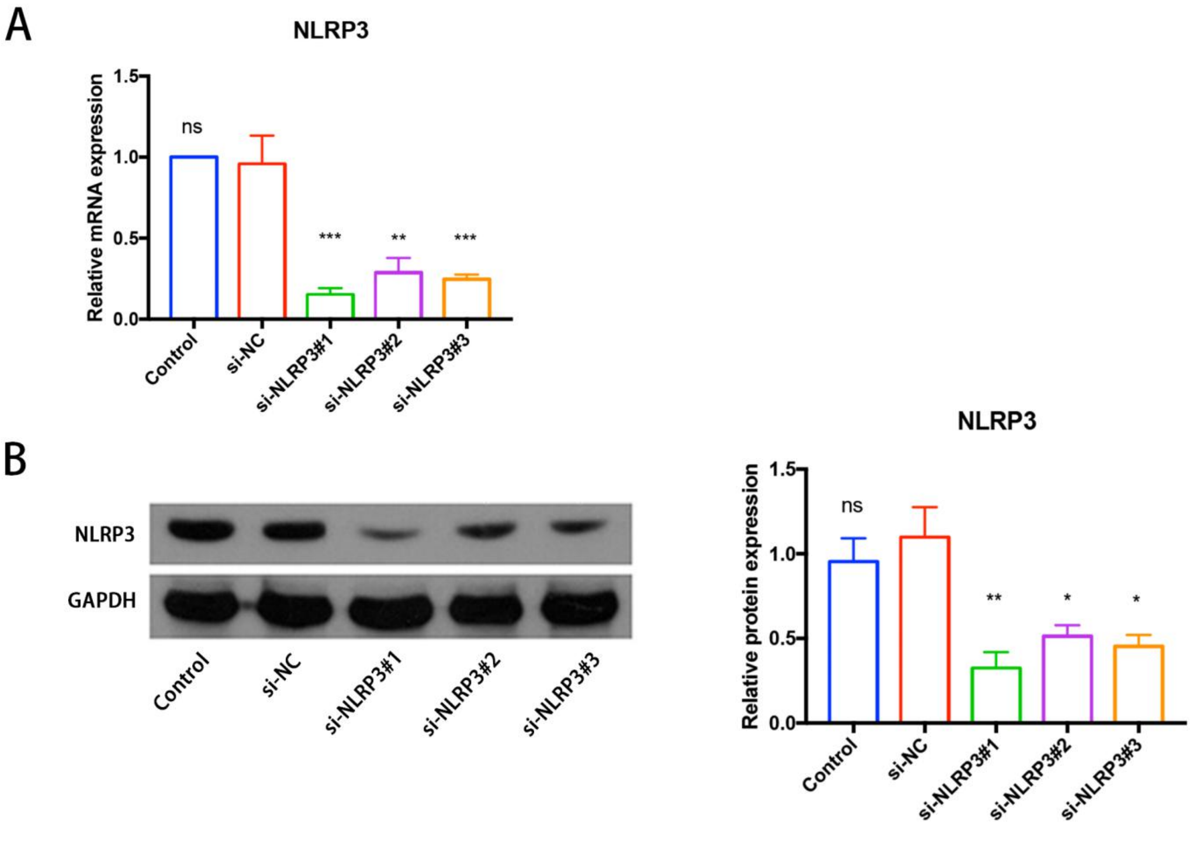

Supplementary Fig 1. The different interfering efficiency of candidate NLRP3-siRNAs. (A) The expression of NLRP3 mRNA after transfection with different candidate NLRP3-siRNA sequence. (B) The expression of NLRP3 protein after transfection with different candidate NLRP3-siRNA sequence. ns: no statistically significance. *p < 0.05, **p < 0.01, ***p < 0.001.

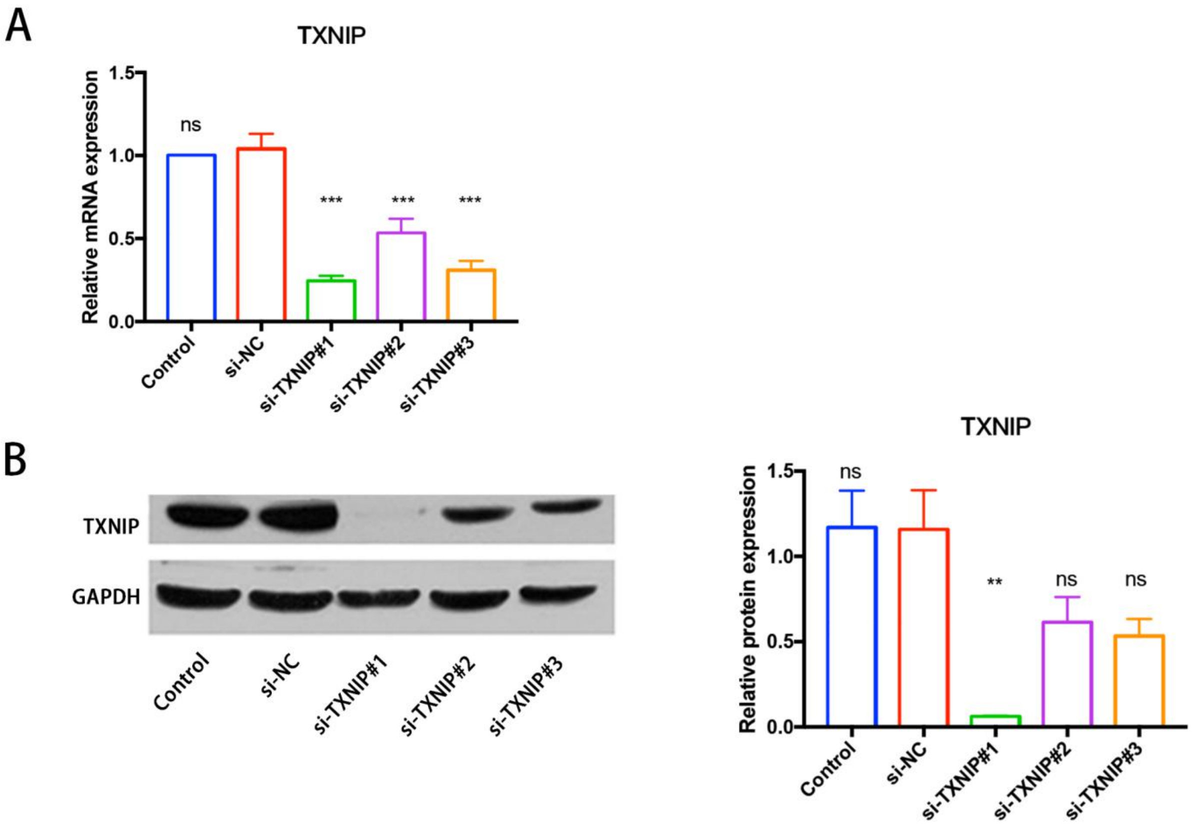

Supplementary Fig 2. The different interfering efficiency of different candidate TXNIP-siRNAs. (A) The expression of NLRP3 mRNA after transfection with different candidate NLRP3-siRNA sequence. (B) The expression of NLRP3 protein after transfection with different candidate NLRP3-siRNA sequence. ns: no statistically significance. *p < 0.05, **p < 0.01, ***p < 0.001.

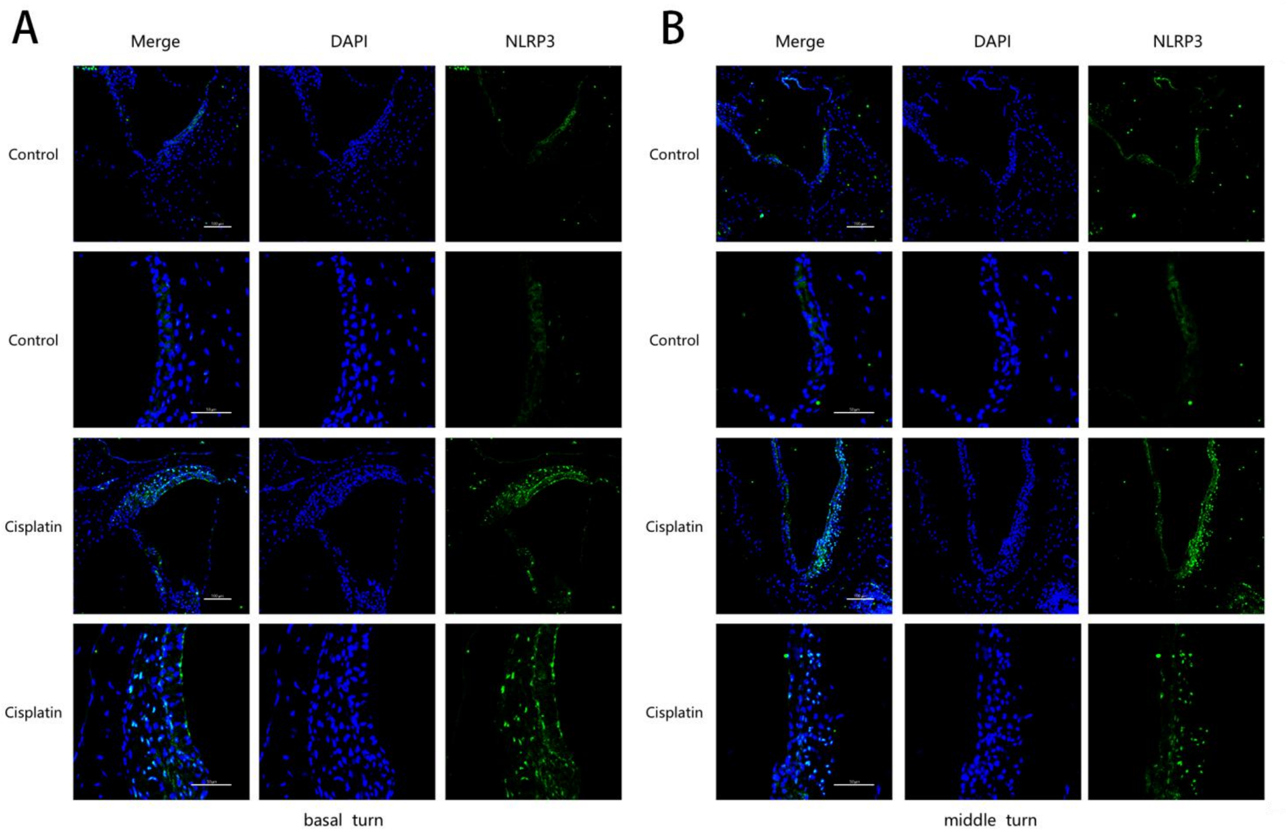

Supplementary Fig 3. Increasing expression in SV of rat cochlear after cisplatin intraperitoneal injection of 4.6 mg/kg × 3d . Immunofluorescence staining in paraffin sections of rat cochlea showed higher expression of NLRP3 in cisplatin SV than control SV. NLRP3 was shown in green and nuclei were stained with DAPI (blue). n=5. The above paraffin sections used for NLRP3 immunofluorescence staining are paraffin sections of the cochlea in the cisplatin-induced ototoxicity rat model prepared in the previous study* of our research group. Scale bar: 50&100μm.

* Liu Tianyi, Zong Shimin#, Luo Pan, Qu Yanji, Wen Yingying, Du Peiyu, Xiao Hongjun: Enhancing autophagy by down-regulating GSK-3beta alleviates cisplatin-induced ototoxicity in vivo and in vitro. Toxicology Letters. 2019, 313C:11-18.
